# Are simple IMRT beams more robust against MLC error? Exploring the impact of MLC errors on planar quality assurance and plan quality for different complexity beams

**DOI:** 10.1120/jacmp.v17i3.6022

**Published:** 2016-05-08

**Authors:** Jiazhou Wang, Xiance Jin, Jiayuan Peng, Jiang Xie, Junchao Chen, Weigang Hu

**Affiliations:** ^1^ Department of Radiation Oncology Fudan University Shanghai Cancer Center Shanghai China; ^2^ Department of Oncology Shanghai Medical College, Fudan University Shanghai China; ^3^ The 1st Affiliated Hospital of Wenzhou Medical College Wenzhou Zhejiang China

**Keywords:** patient QA, planar QA, IMRT

## Abstract

This study investigated the impact of beam complexities on planar quality assurance and plan quality robustness by introducing MLC errors in intensity‐modulate radiation therapy. Forty patients' planar quality assurance (QA) plans were enrolled in this study, including 20 dynamic MLC (DMLC) IMRT plans and 20 static MLC (SMLC) IMRT plans. The total beam numbers were 150 and 160 for DMLC and SMLC, respectively. Six different magnitudes of MLC errors were introduced to these beams. Gamma pass rates were calculated by comparing error‐free fluence and error‐induced fluence. The plan quality variation was acquired by comparing PTV coverage. Eight complexity scores were calculated based on the beam fluence and the MLC sequence. The complexity scores include fractal dimension, monitor unit, modulation index, fluence map complexity, weighted average of field area, weighted average of field perimeter, and small aperture ratio $\left(<5{\text{cm}}^{2}\;\text{and}<50\;{\text{cm}}^{2}\right)$. The Spearman's rank correlation coefficient was calculated to analyze the correlation between these scores and gamma pass rate and plan quality variation. For planar QA, the most significant complexity index was fractal dimension for DMLC (p=−0.40) and weighted segment area for SMLC (p=0.27) at low magnitude MLC error. For plan quality, the most significant complexity index was weighted segment perimeter for DMLC (p=0.56) and weighted segment area for SMLC (p=0.497) at low magnitude MLC error. The sensitivity of planar QA was weakly associated with the field complexity with low magnitude MLC error, but the plan quality robustness was associated with beam complexity. Plans with simple beams were more robust to MLC error.

PACS number(s): 87.55

## I. INTRODUCTION

Planar quality assurance (QA) is a widely used method for patient specific quality assurance in intensity‐modulate radiation therapy (IMRT).^(1)^ An IMRT beam can be labeled as “QA passed beam” or “QA nonpassed beam” by quantitatively analyzing the QA result. A typical planar QA is a two‐step process. First, the planar dose distribution is measured by film or two‐dimensional detector array. Then, the agreement between the calculated and measured planar dose distribution is assessed, using algorithms such as DTA (distance to agreement),^(2)^ DD (dose difference), and gamma analysis.^(3)^ Among these algorithms, gamma analysis is the most widely used algorithm.

The commonly used gamma analysis pass criterion is 90% points on the planar within the region of interest pass gamma criteria of 3%/3 mm.^(4)^ Although this criterion has been used for many years in many institutions, there is no agreement about it^.(5)^ Many studies have tried to find the best cutoff to separate acceptable and unacceptable beams. Carlone et al.^(6)^ provided optimal threshold values with introducing random multileaf collimator (MLC) position errors, which can maximize the sensitivity and specificity of the test by ROC analysis. McKenzie et al.^(7)^ showed that different IMRT QA devices and QA evaluation methods should have different cutoffs after comparing the result of different IMRT QA devices and QA evaluation methods to a “true” IMRT QA result. All these studies assumed that a universal criterion (such as 3%/3 mm, 90%) was suitable for all IMRT beams. However, some studies also pointed out that the complexity of individual beams might relate to delivery accuracy and QA metrics.^4^, ^8^ This means that complex beams with more irregularly shaped apertures and/or smaller segments tend to have lower pass rates in planar QA than simple beams using the same treatment planning system and delivery machine.

To verify this, many innovative methods have been developed to measure plans and beam complexity. The term “beam complexity,” first introduced by Mohan et al.,^(9)^ is characterized by the frequency and amplitude variations of the beam. Webb^(10)^ proposed the modulation index (MI) by measuring variations of photon fluence between neighboring pixels. Fractal dimension was used to assess the level of beam modulation.^(11)^ In the inverse optimization, the complexity metric was used to get a smoother fluence.^(12)^ Some aperture‐based metrics were also developed.^13^, ^14^, ^15^, ^16^

Many studies have investigated the relation between the beam complexity and IMRT QA result, and a weak correlation between beam complexity and gamma pass rate was found.^13^, ^14^, ^15^ These studies were all implemented with clinically commissioned treatment planning systems and clinically commissioned machines. In such circumstances, however, it was difficult to figure out the influence of delivery error on the QA measurement.

To evaluate the effect of MLC error on beams of differing complexity, we introduced MLC errors to the beams. In this study, we explored the sensitivity of planar quality assurance and plan quality to MLC error with varying beam complexity in IMRT. We introduced MLC errors to clinically approved beams and measured the beams' complexity. The impact of MLC errors on different complexity beams and plans was analyzed.

## II. MATERIALS AND METHODS

**Figure 1:**
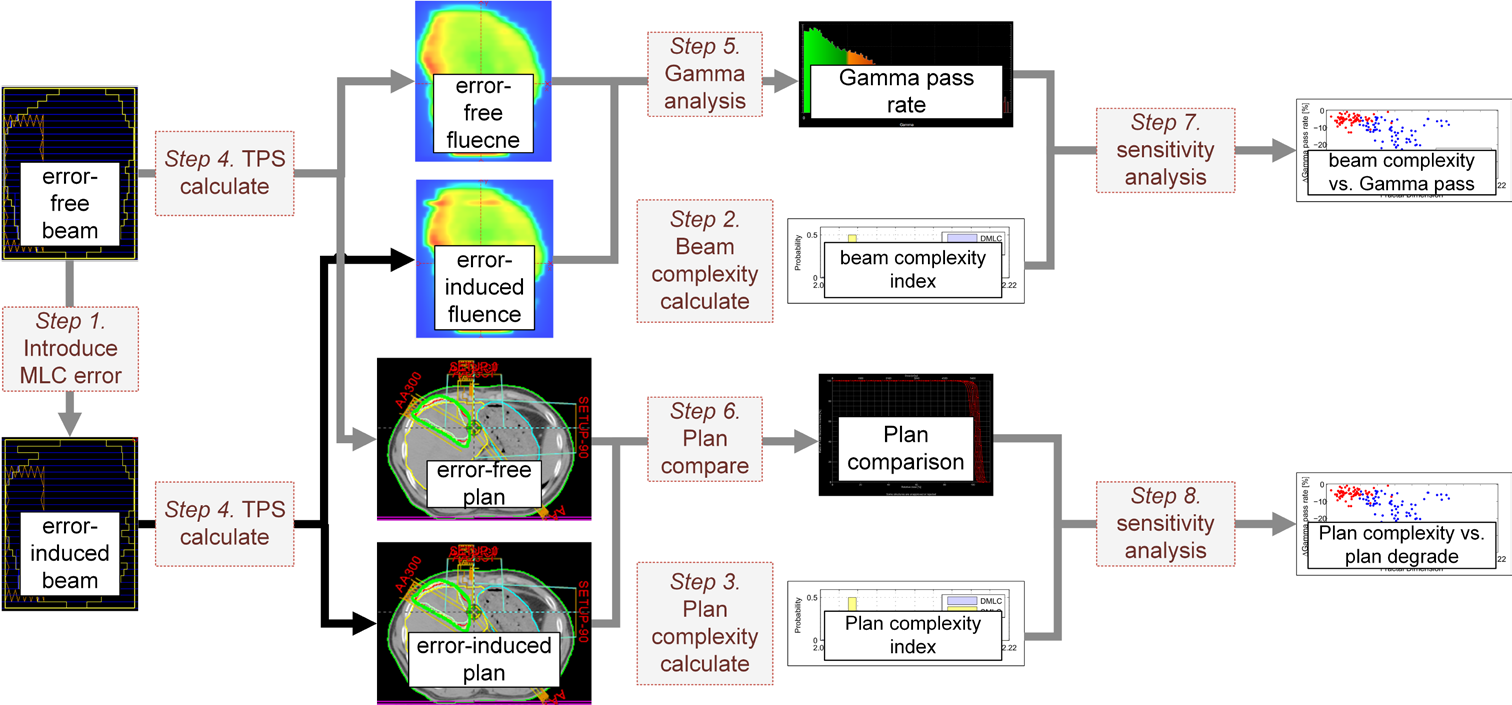
The procedure of the analysis.

The whole procedure is shown in Fig. 1. Dynamic MLC (DMLC) IMRT beams and static MLC (SMLC) IMRT beams were selected from clinically approved plans. First, MLC errors were introduced to generate error‐induced beams (Step 1). Second, the beam complexity and plan complexity were calculated (Steps 2–3). Then, the error‐free and error‐induced fluence maps and plans were calculated from the treatment planning system (TPS) (Step 4). The gamma pass rate, which compares error‐free fluence and error‐induced fluence, was calculated (Step 5). Meanwhile, the plan quality of the error‐free and error‐induced plans was evaluated (Step 6). Finally, we analyzed the relationship between the gamma pass rate and beam complexity (Step 7). The relationship between plan quality variation and plan complexity was also investigated (Step 8).

### A. Plans and base information

Twenty DMLC plans and 20 SMLC plans were enrolled in this study. The basic characters of the beams are listed in Table 1.

The DMLC plans and the fluence maps for DMLC were generated on Eclipse TPS (version 11.0, Varian Medical Systems, Palo Alto, CA). Gamma pass rate was calculated in Portal Dosimetry software (version 11.0, Varian Medical Systems). The parameters for gamma pass rate calculation were set as 1.0% and 1.0 mm, and the region of interest for gamma analysis was set as MLC edge+1.00cm.

The SMLC plans and the fluence map for SMLC were generated on Pinnacle TPS (version 8.0m, Philips Healthcare, Andover, MA). Gamma pass rate was calculated in MATLAB (MathWorks, Natick, MA). The parameters for gamma pass rate calculation were set as 1.0% and 1.0 mm, and the region of interest for gamma analysis was set with a threshold (10% of the maximum dose).

**Table 1:** The basic characters of the beams.

	*DMLC*	*SMLC*
Beam numbers per plan	7.5 [4∼12]	8 [4∼14]
Segments per beam	166 [166∼166]	3.9 [1∼9]
Segment size (cm^2^)^a^	20.94 [4.9∼43.2]	100.4 [31.9∼377.1]
MUs per beam	161.0 [39.9∼472.3]	63.4 [10.0∼206.3]

a The size was measured at isocenter planar.

### B. Error introduce process

To investigate the effect of MLC error on different beam complexities, we introduced artificial delivery error into MLC sequence. The error‐induced plans were generated by MATLAB. For DMLC, The MLC position errors were −0.75, −0.5, −0.25, 0.25, 0.5, and 0.75 mm for each single bank. These artificial errors were similar to the study of Oliver et al.^(17)^ For SMLC, The MLC position errors were −1.50, −1.00, −0.50, 0.50, 1.00, and 1.50 mm for each single bank. After the MLC positions were generated, the plans were reimported into TPS to calculate the error‐induced fluence and dose distribution.

### C. Beam complex calculation

The beam complexity metrics used in this study are listed in Table 2. The complexity index for the whole plan was calculated from the weighted average of beam complexity indices, with the beam MU as the weight factor. The MU index was the total plan MU of all beams.

**Table 2:** The beam complexity metrics.

*Beam Complexity Metrics*	*Description*	*Abbreviation*
Fractal dimension	Gray‐scale algorithm^(28)^	FD
Monitor unit	Monitor unit of beam	MU
Modulation index	The variations of photon fluence between neighboring pixels^(10)^	MI
Fluence maps complexity	Smoothness measurement of in the beam fluence profiles^(12)^	FMC
Weighted segment area	The average segment area for each beam weighted by segment MU	WA
Weighted segment perimeter	The average segment perimeter for each beam weighted by segment MU	WP
Small segment ratio $\left(<5\;{\text{cm}}^{2}\right)$	The ratio of segment area less than defined criteria for each beam^(15)^	${\text{SSR}}_{5}$
Small segment ratio $\left(<50\;{\text{cm}}^{2}\right)$	The ratio of segment area less than defined criteria for each beam	${\text{SSR}}_{50}$

### D. Sensitivity analysis

The gamma pass rate for each magnitude of MLC error was calculated. Spearman's rank correlation coefficient was used to analyze the relationship between the gamma pass rate and beam complexity index. In this study, the plan quality was evaluated using PTV $D_{95}$, which represents the dose covering 95% of PTV. The degree of plan degradation by MLC error was evaluated by the variation of the $D_{95}$, as Eq. (1) describes:

(1) $$E=\frac{D_{95}^{error-induced}-D_{95}^{original}}{D_{95}^{original}}\times 100$$

To summarize the influence of different magnitude errors on gamma pass rate and plan quality, the MLC errors were divided into three groups (low error, medium error, high error) based on the error magnitude. For example, the MLC error with −0.75 mm and 0.75 mm were assigned to the high error group for DMLC. The gamma pass rate and the plan variation were calculated by averaging the errors of same magnitude, as described in Eqs. (2) and (3):

(2) $$\gamma_{passrate}^{high}=\frac{\gamma_{passrate}^{0.75}+\gamma_{passrate}^{-0.75}}{2}$$

(3) $$E^{high}=\frac{E^{0.75}-E^{-0.75}}{2}$$

## III. RESULTS

### A. Gamma pass rate of beams

**Figure 2:**
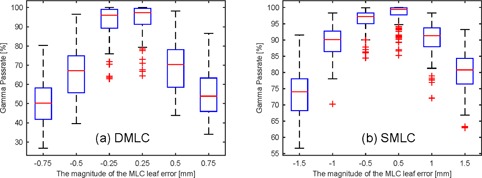
The distribution of gamma pass rate: (a) DMLC beams; (b) SMLC beams.

The distribution of error‐free gamma pass rate and the error‐induced gamma pass rate are shown in Fig. 2. The gamma pass rate decreased with increased MLC leaf error for both DMLC and SMLC. It was clear that most clinical beams' gamma pass rate was over 90% because the low magnitude MLC errors (−0.25 mm and 0.25 mm for DMLC, −0.50 mm and 0.50 mm for SMLC) are more likely to occur in clinical circumstances.

### B. Beams and plans complexity metrics

**Figure 3:**
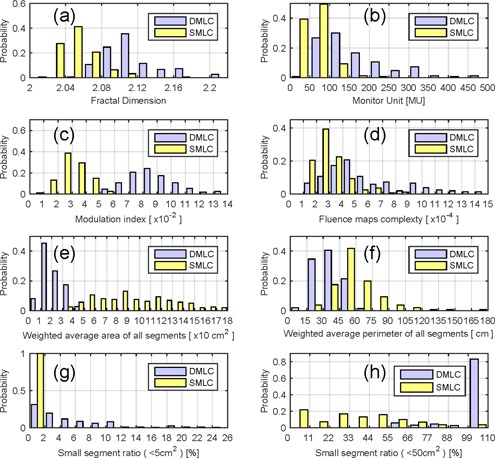
The distribution of complex indices: (a) fractal dimension, (b) monitor unit, (c) modulation index, (d) fluence maps complexity, (e) weighted segments area, (f) weighted segment perimeter, (g) small segment ratio $\left(<5\;{\text{cm}}^{2}\right)$, (h) small segment ratio $\left(<50\;{\text{cm}}^{2}\right)$.

Figure 3 shows the distributions of the complexity metrics. There were no significantly skewed distributions for these complexity metrics, except in ${\text{SSR}}_{5}$ and ${\text{SSR}}_{50}$. For SMLC, the areas of all the segments were larger than 5 cm^2^, while most of DMLC beams' segments were less than 50 cm^2^.

**Figure 4:**
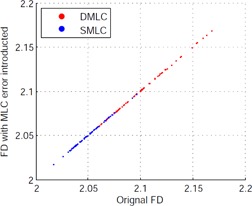
An example of complexity metric variation with MLC error.

To evaluate the stability of the complexity measurement, we calculated the beam complexity metrics in error‐induced beams and compared them to the error‐free beams. We found that none of the complexity measurements were affected by MLC errors. Figure 4 shows an example of the influence of the MLC error to the complexity measurement.

### C. The relation between gamma pass rate and the complexity metrics

**Figure 5:**
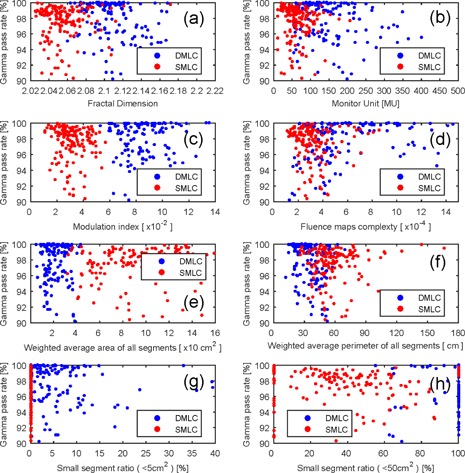
The scatter plot of the gamma pass rate and complex indices for low magnitude MLC error: (a) fractal dimension, (b) monitor unit, (c) modulation index, (d) fluence maps complexity, (e) weighted segments area, (f) weighted segment perimeter, (g) small segment ratio $\left(<5\;{\text{cm}}^{2}\right)$, (h) small segment ratio $\left(<50\;{\text{cm}}^{2}\right)$.

**Figure 6:**
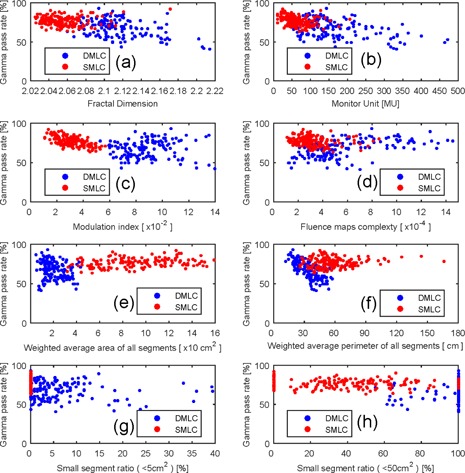
The scatter plot of the gamma pass rate and complex indices for high magnitude MLC error: (a) fractal dimension, (b) monitor unit, (c) modulation index, (d) fluence maps complexity, (e) weighted segments area, (f) weighted segment perimeter, (g) small segment ratio $\left(<5\;{\text{cm}}^{2}\right)$, (h) small segment ratio $\left(<50\;{\text{cm}}^{2}\right)$.

Figure 5 shows the relation between the gamma pass rates and complexity metrics for low magnitude MLC error. Figure 6 shows the relation between the gamma pass rates and complexity metrics for high magnitude MLC error. Table 3 shows the correlation coefficient between gamma pass rate and MLC error. Many complexity metrics were related to gamma pass rate. For DMLC, the most related complexity index was WP at medium and high magnitude MLC error. The FD was the most related complexity index at low magnitude MLC error. For SMLC, the most related complexity index was MI at high magnitude MLC error, FMC at medium magnitude MLC error, and WA at low magnitude MLC error.

**Table 3:** The correlation coefficients of gamma pass rate and complexity indices.

	*DMLC*			*SMLC Medium*		
*Beam Complexity Metrics*	*Low*	*Medium*	*High*	*Low*	*Medium*	*High*
Fractal dimension	−0.40 ^a^	−0.33 ^a^	−0.33 ^a^	−0.01	0.01	−0.13
Monitor unit	−0.03 ^a^	0.12^a^	0.16^a^	−0.18 ^b^	−0.24 ^b^	−0.39 ^a^
Modulation index	−0.03	0.12	−0.37 ^b^	−0.17 ^b^	−0.20 ^b^	−0.50 ^a^
Fluence map complexity	0.30^a^	0.50^a^	0.57^a^	−0.21 ^b^	−0.31 ^a^	−0.27 ^a^
Weighted segment area	0.11	−0.13	−0.22 ^b^	0.27^a^	0.30^a^	0.37^a^
Weighted segment perimeter	−0.37 ^a^	−0.55 ^a^	−0.63 ^a^	−0.01	−0.07	0.07
Small segment ratio $\left(<5\;{\text{cm}}^{2}\right)$	−0.24 ^b^	−0.06	−0.01	‐	‐	‐
Small segment ratio $\left(<50\;{\text{cm}}^{2}\right)$	‐	‐	‐	−0.24 ^b^	−0.17 ^b^	−0.16 ^b^

a Significant complexity score (p= <0.001).

b Significant complexity score (p= <0.05).

### D. The relationship between plan quality degrade and the complexity metrics

**Figure 7:**
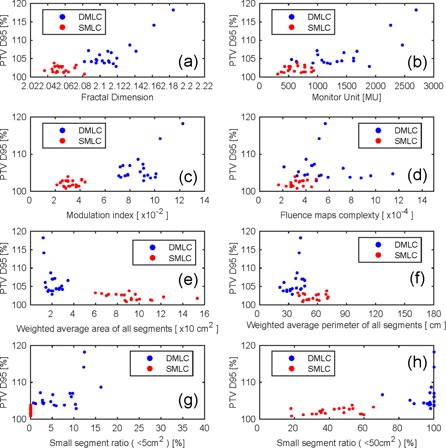
The $D_{95}$ variation and complex indices for high magnitude MLC error: (a) fractal dimension, (b) monitor unit, (c) modulation index, (d) fluence maps complexity, (e) weighted segments area, (f) weighted segment perimeter, (g) small segment ratio $\left(<5\;{\text{cm}}^{2}\right)$, (h) small segment ratio $\left(<50\;{\text{cm}}^{2}\right)$.

Figure 7 shows the relationship between the variation of the $D_{95}$ and complexity metrics for high magnitude MLC error. Table 4 shows the correlation coefficient between $D_{95}$ variation and MLC error. Many complexity metrics were related to $D_{95}$ variation. For DMLC, the most related complexity index was WP with all magnitudes of MLC error. For SMLC, the most related complexity index was MI at high and medium magnitude MLC error. WP was the most related complexity index at low magnitude MLC error.

**Table 4:** The correlation coefficients of the PTV D95 variation and complexity indices.

	*DMLC*			*SMLC*		
*Beam Complexity Metrics*	*Low*	*Medium*	*High*	*Low*	*Medium*	*High*
Fractal dimension	0.45^a^	0.44	0.43	0.20	0.12	0.07
Monitor unit	0.48^a^	0.45^a^	0.44	0.43	0.64^a^	0.67^a^
Modulation index	0.06	0.11	0.09	0.11	0.11	0.10
Fluence map complexity	−0.31	−0.27	−0.27	0.38	0.29	0.29
Weighted segment area	−0.20	−0.23	−0.24	−0.49 ^a^	−0.52 ^a^	−0.51 ^a^
Weighted segment perimeter	0.56^a^	0.52^a^	0.51^a^	−0.01	0.23	0.24
Small segment ratio $\left(<5\;{\text{cm}}^{2}\right)$	0.34	0.33	0.34	‐	‐	‐
Small segment ratio $\left(<50\;{\text{cm}}^{2}\right)$	‐	‐	‐	0.31	0.52^a^	0.57^a^

a Significant complexity score (p= <0.05).

## IV. DISCUSSION

In this study, we analyzed the sensitivity of the planar QA and plan quality to different magnitudes of MLC error with different beam complexities. Our results showed that some complexity indices could reflect the sensitivity of beams to MLC error (p<0.05). Furthermore, some indices (for example the WP and FMC in DMLC at high magnitude MLC error) were significantly related to the gamma pass rate (p<0.001). Meanwhile, the Spearman's correlation coefficients of these indices were about 0.5, which usually represent a moderate correlation.^(18)^ For DMLC, most correlation coefficients were increased with MLC error magnitude for FMC and WP. At low magnitude MLC error, the FD and WP have similar correlation coefficients (−0.40 and −0.37). Because a low magnitude MLC error is more likely than a high one in clinical circumstances, the FD and WP may be the appropriate indices to evaluate the beam complexity for DMLC. For SMLC, many indices were significantly related to the gamma pass rate. However, at the low and medium magnitude MLC error, the Spearman's correlation coefficients for the most significant indices were about 0.3, which usually represent a negligible correlation.^(18)^

The correlation between plan complexity and plan quality variation was also investigated. For DMLC, we found that the FD, MU, and WP were significantly related to the plan quality variation. The Spearman's correlation coefficients were roughly the same for different magnitudes of MLC errors. For SMLC, we found that the WA, MU, and ${\text{SSR}}_{50}$ were significantly related to the plan quality variation. These complexity metrics were more sensitive to high magnitude MLC error than low magnitude MLC error.

Some studies have similar results. McGarry et al.^(13)^ reported there was a trend toward fewer failed pixels with decreased complexity. Nauta et al.^(11)^ reported a lower gamma pass rate for high modulate field described by FD. Meanwhile McNiven et al.^(14)^ and Du et al.^(16)^ reported no correlation between the IMRT gamma pass rate and complexity metrics. As our study showed, different magnitude of MLC error and different complexity metrics may have different results. The differing results between these studies can be explained by the different magnitudes of MLC errors explored in these studies.

Theoretically, the degradation of the beam's gamma pass rate is mainly caused by two factors. The first factor is the TPS calculation, which includes the beam modeling and the accuracy of the dose engine. For example, the small field may not be perfectly modeled.^(19)^ This will cause the beam with smaller aperture to have more uncertainties. The second factor is related to machine delivery, including the accuracy of MLC position and the dose rate accuracy. In our study, we only concerned about the MLC position error.

IMRT planar QA is a time‐consuming and potentially inaccurate method.^(20)^ Some studies showed the result of planar QA could not predict clinically relevant patient dose error.^21^, ^22^, ^23^, ^24^ To overcome the shortcomings of planar QA, some new patient‐specific QA methods have been developed.^25^, ^26^, ^27^ As technology develops, planar QA may be replaced or updated in the future.

## V. CONCLUSIONS

The sensitivity of gamma pass rate is related to some complexity metrics. The impact of the MLC errors to the planar QA result is weak at low magnitude MLC error. The variation in plan quality is also related to plan complexity for both DMLC and SMLC.

## COPYRIGHT

This work is licensed under a Creative Commons Attribution 4.0 International License.

## Supporting information

Supplementary Material

Supplementary Material
