# Supplementary figures and images for: Are simple IMRT beams more robust against MLC error? Exploring the impact of MLC errors on planar quality assurance and plan quality for different complexity beams
Source: J Appl Clin Med Phys. 2016 May 8;17(3):147–57. doi: 10.1120/jacmp.v17i3.6022 (PMC5690928; doi:10.1120/jacmp.v17i3.6022)

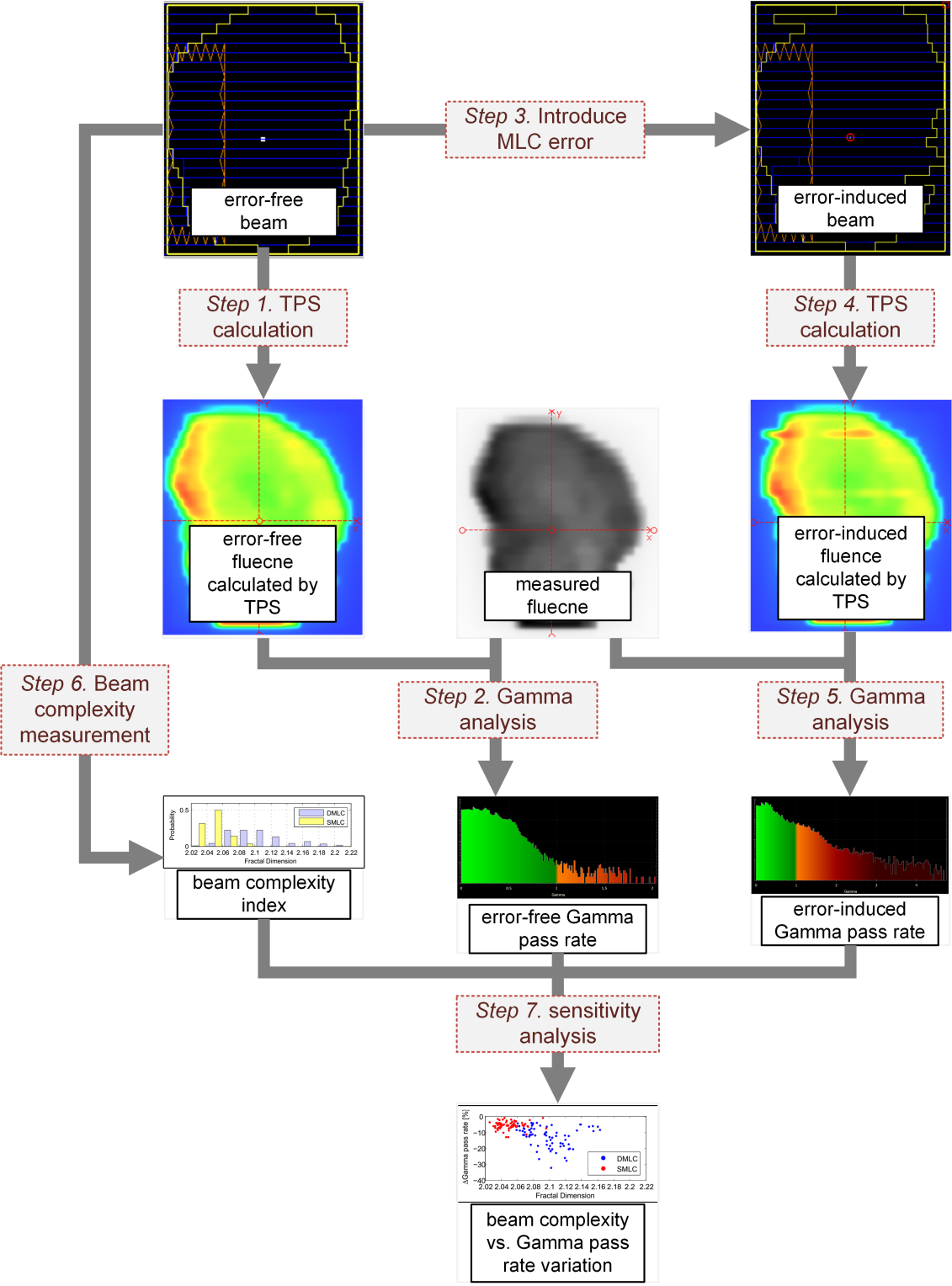

Supplement: Supplementary file 1 — Supplementary Material [file ACM2-17-147-s001.png]

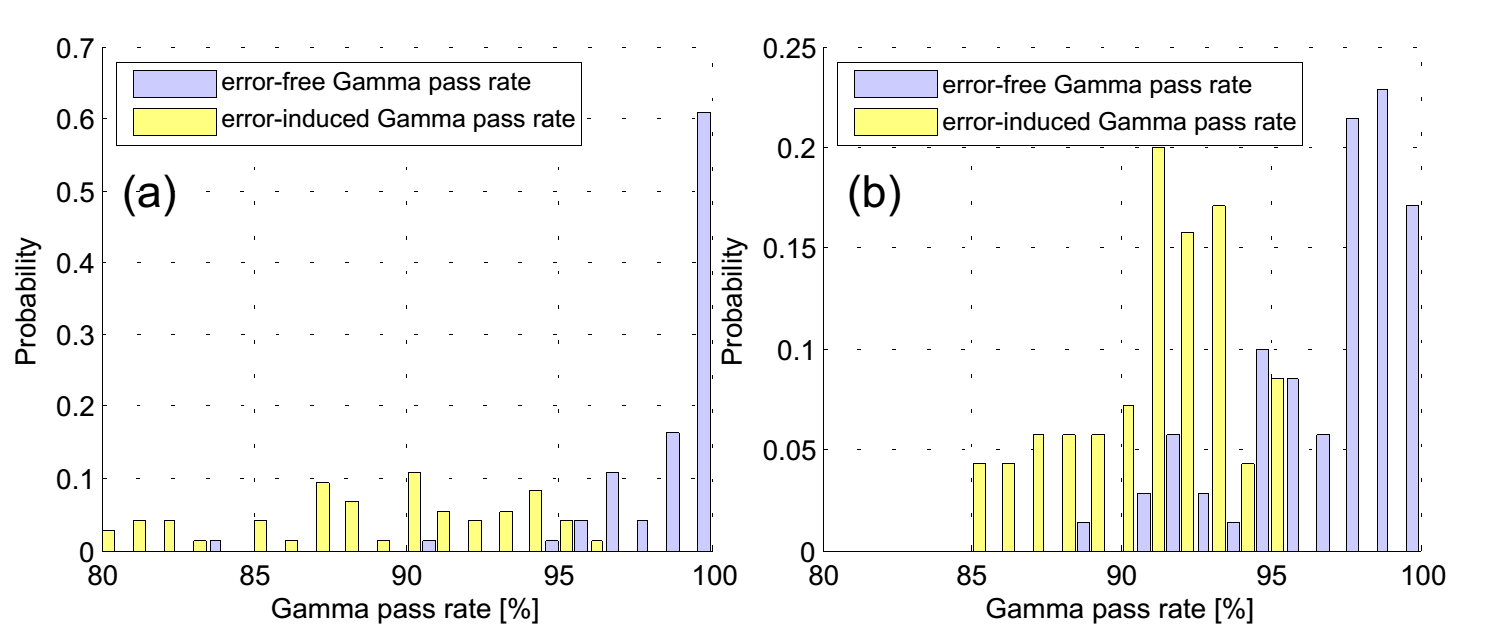

Supplement: Supplementary file 2 — Supplementary Material [file ACM2-17-147-s002.png]
